# Competing demands in postpartum care: a national survey of U.S. providers’ priorities and practice

**DOI:** 10.1186/s12913-020-05144-2

**Published:** 2020-04-06

**Authors:** Tamar Krishnamurti, Hyagriv N. Simhan, Sonya Borrero

**Affiliations:** 1 Division of General Internal Medicine, University of Pittsburgh, 200 Meyran Avenue, Suite 200, Pittsburgh, PA 15213 USA; 2 Magee-Womens Research Institute, Department of OB-GYN and Reproductive Sciences, University of Pittsburgh, Pittsburgh, PA 15213 USA; 3 Division of General Internal Medicine, University of Pittsburgh, Pittsburgh, PA 15213 USA; 4 Center for Health Equity Research and Promotion, VA Pittsburgh Healthcare System, Pittsburgh, PA 15213 USA

**Keywords:** Postpartum, Clinical care guidelines, Medical decision-making, Telemedicine, Intimate partner violence, Depression, Pelvic exam, Maternal mortality, ACOG

## Abstract

**Background:**

A new movement towards improved postpartum care calls for restructuring how that care is provided. As professional guidelines evolve, understanding how providers *prioritize* and *practice* postpartum care can offer insights about elements of care that may be currently performed more routinely than providers deem a priority to do so, as well as those that may warrant more routine practice.

**Methods:**

We surveyed 600 randomly-sampled U.S. postpartum care providers about their priorities for and the frequency with which specific elements of care are provided, as well as the feasibility of remote delivery of postpartum care provision (i.e., telemedicine).

**Results:**

The survey response rate was 43% across medical specialties. Providers reported an average of only 24.4 ± 11.7 min available to spend on the postpartum visit. Certain types of postpartum care were highly prioritized and routinely performed, such as depression screening. Yet, there were also noted discrepancies between prioritized and performed care, revealing competing demands on providers’ time. For example, pelvic exams were performed more often than similarly prioritized care, whereas screening for intimate partner violence and substance use were performed less often than similarly-prioritized care. Certain types of care were identified as important that are not explicitly addressed by national practice guidelines (e.g. transitioning to parenthood). Approximately 25% of respondents regarded telemedicine as a feasible remote care delivery alternative to much of the care currently provided in-person.

**Conclusions:**

The time providers have available to offer comprehensive postpartum care is constrained. Understanding how certain elements of care may be competing with one another at a single postpartum visit highlights those elements of care, which may be currently underperformed, as well as those elements of care that may warrant evaluation for future inclusion in standard care. For some providers experiencing time constraints, complementary remote care represents a potentially viable approach to implementing recommendations for transitioning the traditional visit to a more frequent, ongoing postpartum care process. In calling for a new approach to postpartum care delivery, professional organizations should consider the practices and priorities of their constituency as they revise guidelines and shape future research about the value of specific postpartum services.

## Background

The traditional practice of a single postpartum appointment 6 weeks after birth has lately been called into question. The American College of Obstetricians and Gynecologists (ACOG) published new guidelines in 2018 specifically recommending postpartum care “become an ongoing process, rather than a single encounter, with services and support tailored to each woman’s individual needs” [1]. The guidelines were, in part, published in response to the increasing U.S. maternal mortality rate [2], which stand in contrast to global maternal mortality patterns [3]. Maternal mortality review committees report that over 60% of U.S. maternal deaths may have been prevented with more timely diagnoses and effective treatment for postpartum onset conditions, as well as improved patient knowledge of warning signs [4]. Furthermore, research has demonstrated the majority of maternal deaths occur in the 42 days following birth [5], with approximately 25% occurring after women are discharged from the hospital following pregnancies and deliveries that appear uncomplicated. Pregnancy-related deaths are highest among non-Hispanic black women, women at older ages [6], and those with public health insurance [7]. Thus, traditional practices may result in a lack of care during a critical time, particularly for those women at baseline higher risk of maternal mortality.

Yet the ways in which postpartum care should be prioritized and structured for each individual woman remains an open question, particularly as ACOG suggests that some postpartum care assessments need not occur as an in-person office visit, but could be completed through various forms of remote monitoring. Existing guidelines for postpartum appointment care provision vary in their scope and detail [8] and there is not always a clear evidence-base for the value and timing of performing the current elements of in-person postpartum care. As such, postpartum care providers, and the healthcare systems in which they work, face the challenge of determining what elements of care to deliver at each postpartum care visit, how many visits are appropriate, and how to tailor that care to the individual patient’s needs at each of those visits. As postpartum care guidelines evolve, professional organizations have an opportunity to revisit standard care elements, helping to clarify these open questions, and supporting informed decision making on the part of providers.

Before determining how and when various elements of postpartum care should be administered as part of an ongoing postpartum period of care, however, it is critical to understand how providers prioritize and perform the elements of postpartum care that are currently dictated by (or absent from) existing guidelines. By examining the priorities and practices among a national sample of postpartum healthcare providers, we can begin to identify what aspects of these guidelines may warrant further examination, either because valuable elements of care are currently underperformed or because the value of certain care elements is unclear. To this end, we administered a survey to a national sample of postpartum care providers, asking about their postpartum care practice. We analyze the tradeoffs that providers may make to lend insight into which elements of care those providers may interpret as less valuable or find more challenging to administer given their time constraints. We also explore providers’ support for and capability of performing this care using remote (telemedicine) means.

## Methods

### Study sample and recruitment

The mailing addresses of 6000 active obstetrician-gynecologist (OB-GYN) physicians, 3000 active family medicine physicians and 6542 active nurse-midwives were acquired from the official databases of three professional organizations: The American Medical Association (AMA), The American Academy of Family Physicians (AAFP) and The American College of Nurse-Midwives (ACNM). From each database sample, 200 healthcare providers were randomly selected to receive a mailed survey directly from the researchers.

Professional mailing addresses were verified with a Google search. Paper surveys were mailed in March 2018–May 2018. Each mailing contained a paper survey and prepaid return envelope, a cover letter with a $10 bill for participation, and a QR code for optional online survey completion. The survey was developed specifically for this study and a copy of all survey questions may be found in supplemental materials (Additional File S1). Surveys were formatted following Dillman’s Total Design Method to improve validity and response rate [9]. Formatting included hand writing the mailed envelopes, using individual stamps rather than prepaid envelopes, personalizing the cover letters with the clinician’s title, and providing multiple options for completing the survey (online and paper). Three weeks after the initial contact was sent, a short letter was mailed reminding clinicians to complete the survey. Participants whose mail was returned to sender or who returned an uncompleted survey were not included in the follow-up mailing.

### Survey measures

Before distributing the survey, semi-structured phone and in-person cognitive interviews were completed with 8 postpartum healthcare providers to refine all questions and survey content.

The survey presented questions in a number of formats (binary choice, multiple choice, Likert-scale ratings, and open-ended) about elements of postpartum care that were identified from three national professional organizations [10, 11], as well as HEDIS [12], and WHO [13] guidelines.

Respondents rated, on a 5-point Likert-scale, their priorities for postpartum care (*How important is it to address each of the following at an in-office 6-week postpartum visit?*), as well as the frequency of practice of the same elements (*How often do you address each of the following at an in-office 6-week postpartum visit?*). Categories included: Clinical Elements (vaginal birth complications; C-section complications; physical/pelvic exam; pregnancy onset complications (e.g., hypertension); nonpregnancy related chronic conditions (e.g., cardiovascular disease); and transitioning to primary care), Family Planning (contraceptive counseling and family planning, contraceptive provision, and resuming sexual activities), Behavioral Health (postpartum depression and other mental health issues, intimate partner violence and other safety issues, healthy maternal sleep, weight trajectory and diet information, smoking, and opioid and other substance abuse) and Infant Care (breastfeeding and other infant feeding issues and safe sleep). Respondents could also add their own priority elements and ratings in an open-ended response option.

Providers were also asked about the optimal timing for postpartum care (multiple choice with optional open-ended response), the main reasons a patient should attend a routine postpartum appointment (open-ended), characterization of their care provision relationship (multiple choice), and average amount of time spent providing care (in minutes). Finally, providers reported on the feasibility of performing telemedicine in their current practice immediately or at some point in the future (multiple-choice) and whether aspects of the postpartum visit could be assessed as effectively by telemedicine as by an in-person visit (yes/no and open-ended explanation).

### Statistical analysis

All paper survey responses were double-entered. All demographic variables and questionnaire responses were summarized by medical specialty (OB-GYN, Family Medicine, and Nurse Midwives) using descriptive statistics (mean and standard deviation for continuous measures; count and percent for categorical measures). We compared differences between practitioner groups using t-tests, ANOVA, and Chi-squared tests. All open-ended responses were coded by two independent coders for thematic content and a Cohen’s kappa was calculated to measure inter-rater reliability.

To quantify discrepancies in care priorities and actual practice, the effect size (Cohen’s d) was calculated for differences in ratings. To ensure that effects were not attributable to different use of the two scales (importance and frequency), both scales were normalized for analysis. Elements for which frequency had a higher mean score than importance, or vice versa, were considered to be ‘inefficiencies.’ Elements with comparable importance scores, but differences in performed frequency, were considered instances in which providers may potentially be forced to make ‘tradeoffs’ in care.

The statistical software package SPSS 25.0 (SPSS Inc., Chicago, IL) was used for all data analyses.

### Consent to participate

This study was approved by the University of Pittsburgh Institutional Review Board in November 2017 under the protocol number 17100584. The need for consent was waived for this study by the University of Pittsburgh Institutional Review Board.

## Results

### Respondents

Of the 600 surveys sent to providers (200 per specialty subgroup), 50 were excluded from analysis. Of the 50 excluded, 29 surveys were returned to sender as an undeliverable address (55% were from the AMA list, 21% were from the ACNM list, and 24% were from the AAFP list) and 21 recipients returned an uncompleted survey due to being retired or having a non-relevant specialty (over 50% of these were from the AAFP list). A total of 20 surveys were returned uncompleted and in their original envelope with no explanation. To be conservative, these 20 surveys were categorized as refusals, rather than undeliverable, and were included in response rate analyses.

Based on eligible responses, the overall response was rate 43% (236/550). The majority - 72% (170/236) - returned the survey by mail. Table 1 provides respondents’ demographic data and their characterization of their patient population by provider specialty. Region was determined from the respondent’s reported primary practice zip code.

**Table 1:** Provider Respondent Characteristics and Patient Population

Characteristics	Frequency (%)			
	All respondents (*n* = 236)	Nurse-Midwives (*n* = 106)	Family Medicine (*n* = 63)	OB-GYN (*n* = 62)
**Mean years in practice**	25.0 ± 11.8	22.8 ± 12.7	28.8 ± 9.7	24.1 ± 11.2
**Actively treating postpartum patients**	214 (90.7%)	98 (92.5%)	52 (82.5%)	60 (96.8%)
**Gender**				
Female	159 (67.4%)	104 (98.1%)	28 (44.4%)	26 (41.9%)
Male	75 (31.8%)	1(0.9%)	34 (54.0%)	36 (58.1%)
**Race**				
Asian	12 (5.1%)	1 (0.9%)	4 (6.3%)	6 (9.7%)
Black/African American	11 (4.7%)	5 (4.7%)	1 (1.6%)	5 (8.1%)
Hispanic/Latino(a)	8 (3.4%)	2 (1.9%)	2 (3.2%)	4 (6.5%)
Native American	0 (0%)	0 (0%)	0 (0%)	0 (0%)
Mixed race, Other	5 (2.1%)	0 (0%)	3 (4.8%)	2 (3.2%)
White/Caucasian	197 (83.5%)	97 (91.5%)	51 (81.0%)	45 (72.6%)
Did not respond	3 (1.3%)	1 (0.9%)	2 (3.2%)	0 (0%)
**Region of Practice**				
Northeast	39 (16.5%)	26 (24.5%)	3 (4.8%)	10 (16.1%)
Midwest	72 (30.5%)	23 (21.7%)	29 (46.0%)	17 (27.4%)
South	68 (28.8%)	33 (31.1%)	13 (20.6%)	21 (33.9%)
West	53 (22.5%)	21 (19.8%)	17 (27.0%)	14 (22.6%)
**Proportion of patients on Medicaid**	42.9 ± 29.4	50.9 ± 30.1	37.7 ± 28.0	35.7 ± 27.5
**Postpartum care attendance**				
Schedule care	90.4 ± 14.1	90.1 ± 13.2	87.7 ± 20.4	93.0 ± 8.6
Attend care	79.4 ± 18.8	79.1 ± 18.4	75.6 ± 23.7	82.2 ± 14.9

### Priorities for care

Table 2 shows the mean Likert-scale–rated priorities for each postpartum care element compared with mean reported frequency of practice of that element across provider types. It also shows the effect size of the difference in priority and practice (Cohen’s d), which appropriately adjusts for non-normal distribution.

**Table 2:** Cohen’s d of Importance-Rank Difference for Postpartum Care Categories

Categories	Importance Mean (SD)^a^	Frequency Mean (SD)^b^	Cohen’s d^c^
**Clinical Elements**			
C-section birth complications	4.51(.73)	4.70(.68)	0
Vaginal birth complications	4.47(.77)	4.74(.64)	0
Pregnancy-related complications	4.32(.80)	4.57(.75)	.1
Chronic health conditions	3.76(.91)	3.98(.93)	.1
Transitioning to primary care	3.39(1.17)	3.33(1.35)	.1
Physical/pelvic exam	3.28(1.10)	4.08(1.05)	**.7**^**d**^
**Behavioral**			
Depression	4.78(.41)	4.90(.46)	.1
Intimate partner violence	4.32(.78)	3.90(1.05)	**.6**
Substance use	4.19(.88)	3.78(1.17)	**.5**
Smoking	4.13(.85)	4.01(1.11)	.2
Maternal sleep	3.98(.81)	3.92(.99)	.2
Diet and weight trajectory	3.53(.92)	3.62(.97)	0
**Family planning**			
Family planning counsel	4.63(.61)	4.89(.49)	.2
Contraceptive provision	4.52(.68)	4.59(.81)	.1
Resuming sexual activity	3.96(.85)	4.70(.65)	**.8**^**d**^
**Infant Health**			
Breast health, breastfeeding and other infant feeding issues	4.45(.71)	4.66(.66)	.1
Infant safe sleep	3.70(1.10)	3.38(1.30)	.3

^*a*^ Importance scale ranged from “1 = not at all” to “5 = extremely,” with a midpoint of “3 = moderately.”

^b^ Frequency scale ranged from “1 = never” to “5 = always,” with a midpoint of “3 = sometimes.”

^c^ Cohen’s d calculations were performed on differences between importance and frequency on normalized scales. Medium (Cohen’s d values > .5) or Large (Cohen’s d values > .8) differences highlighted in bold.

^d^ Indicates element is performed more frequently than it is prioritized.

In terms of specific elements of care, there was generally high correspondence between prioritized and performed care. For example, depression screening was an element that was both highly prioritized and frequently performed, as was addressing birth-related and pregnancy-onset complications. There were, however, a few large potential discrepancies in care across all specialties, identified by calculated normalized differences in priority and practice: the pelvic exam, counseling regarding resumption of sexual activity, and intimate partner violence screening. The first two elements were performed *more* frequently than the level at which they were prioritized, pointing to inefficiencies in care. Intimate partner violence screening, on the other hand, was performed *less* often than would be expected considering its perceived importance, perhaps pointing to a potential tradeoff being made given competing demands on time.

Several elements were prioritized and performed differently depending on provider type. For example, an ANOVA identified that, on a 5-point Likert scale, Family Medicine physicians and Nurse-Midwives both prioritized infant safe sleep education provision (4.07 ± 0.96 and 3.79 ± 1.10, respectively) more highly than OB-GYNs (3.31 ± 1.05), *F* (2, 224) *=* 8.03*, P* < .001, and performed it more frequently, *F* (2, 2224) *=* 20.23*, P* < .001. While there were no significant differences in the high priority placed on opioid and other substance use counseling across provider types, it was more routinely provided by Family Medicine physicians (4.17 ± 1.02), than by Nurse-Midwives (3.77 ± 1.18) or OB-GYNs (3.5 ± 1.19), *F* (2, 209) *= 5.18, P* < .01.

**Fig. 1:**
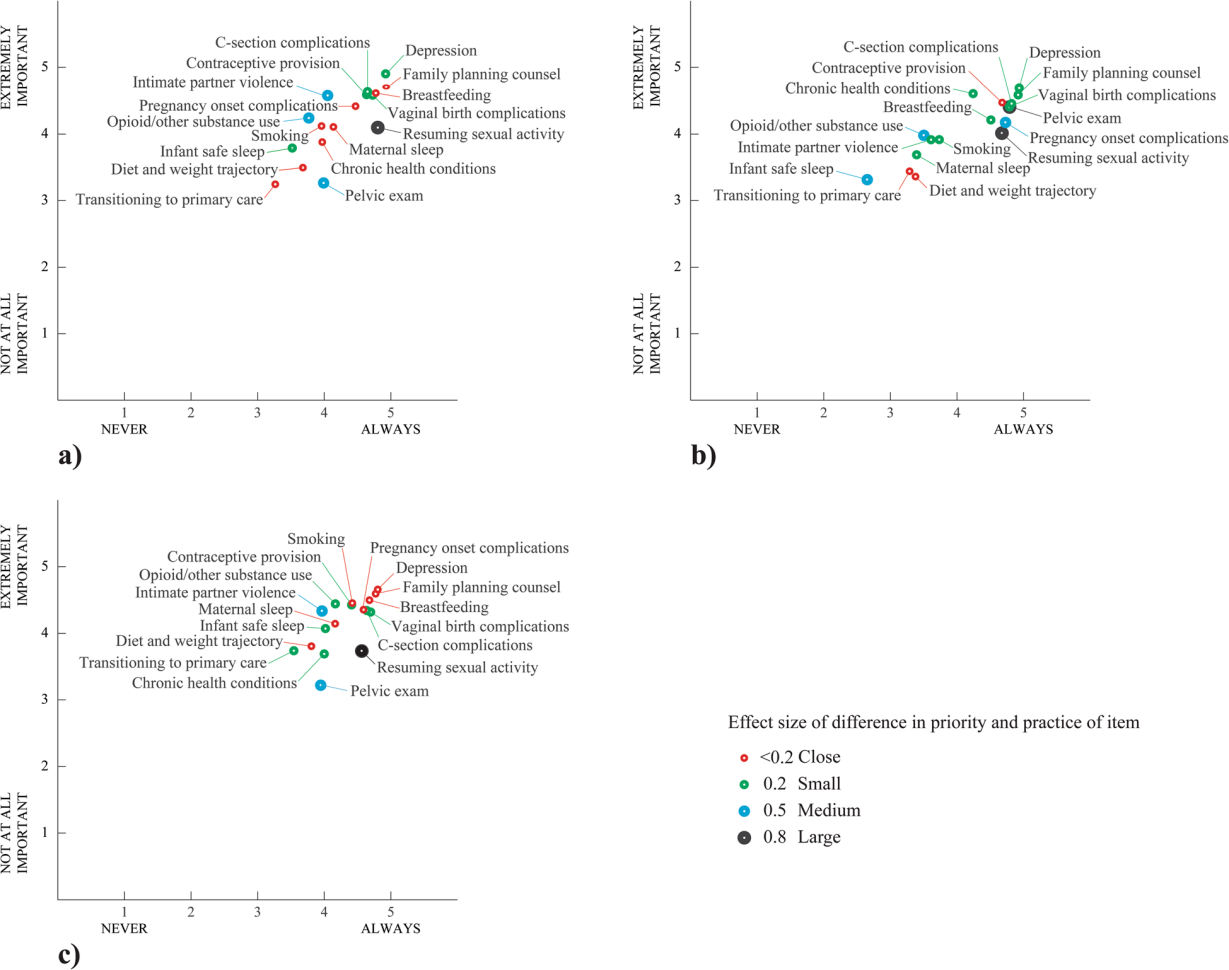
Likert-scale rated priorities for postpartum care contrasted against reported frequency of practice for (**a**) OB-GYN respondents (**b**) Family Medicine respondents and (**c**) Nurse-Midwife respondents

Figure 1 illustrates Likert-scale–rated priorities for postpartum care compared with reported frequency of practice broken down by provider type for (a) OB-GYN (b) Family Medicine and (c) Nurse-Midwife respondents.

Elements that show equivalent numeric ratings for priority but differ in performed frequency can be conceptualized as competing demands under time constraints, whereby some elements of care are routinely “traded-off” against other forms of care. Identifying such tradeoff elements can lend insight into prioritization and distribution of postpartum care. As mentioned above, counseling regarding the resumption of sexual activity can be broadly considered inefficient. For Nurse-Midwives, it was performed more frequently than equivalently prioritized maternal sleep assessment and smoking cessation counseling. For OB-GYNs, on the other hand, it was performed more frequently than equivalently prioritized smoking cessation counseling, opioid and other substance use assessment, and intimate partner violence screening. In the case of Family Medicine physicians, it was performed more frequently than equivalently prioritized discussions about transitioning to primary care and discussion of chronic health conditions. By the same token, screening for intimate partner violence was consistently underperformed relative to its judged priority. For both Family Medicine physicians and Nurse-Midwives, this was specifically in contrast with vaginal birth and C-section complications, which were both performed more frequently.

Additional postpartum care elements, not included in current guidelines, but that were seen as important by all provider types (based on coding of open-ended text reports of care) largely fell into a category that could be called, “Transition to Parenthood.” This included certain aspects involved in evaluating the social, emotional, and tangible support available to patients as they transition to motherhood, including family relationships and their work environment. Providers also identified other important care elements, such as infant bonding and vaccine schedules, as well as reviewing a woman’s birth experience and planning for future pregnancies. Inter-rater reliability analysis found moderate agreement between the two raters after the first round of coding for this open-ended response (Kappa = 0.79, *P* < .001).

### Characterization of care

Providers largely favored earlier care with 37.7% preferring a single in-person visit within 1 to 3 weeks postpartum and 19.5% wanting both earlier and more frequent care. Many (31.4%) responded that the traditional 6-week postpartum visit was most effective. Only a small percentage specified that a later than 6-week visit would be most effective (8.9%) or indicated a postpartum visit only be required if specific concerns needed to be addressed (2.1%). A small percentage (11.4%) provided open-ended comments about their postpartum timing preference. From these comments, the primary reasons offered for earlier postpartum appointments were timelier intervention for delivery-related complications, mental health, breastfeeding, and contraceptive needs, all of which may present challenges to the patient earlier than 6-weeks’ postpartum. Some also mentioned providing schedule flexibility to patients (e.g. allowing patients to coordinate the postpartum visit with their infant’s first well-visit).

Providers reported an average of 24.4 ± 11.7 min spent with each patient at their postpartum visit. An ANOVA identified significant differences in time allotted depending on specialty; Nurse-Midwives (28.1 ± 12.7 min) and Family Medicine providers (25.1 ± 10.9 min) reported more time than OB-GYNs (17.6 ± 6.9 min), *F* (2, 216) = 17.51*, P* < .001.

A chi-squared analysis identified that the nature of care provision differed significantly by provider type, according to reports from each, *X*^2^ (8, *N* = 224) = 19.89, *p* = .002. Almost all respondents routinely provided postpartum care (> 90%). Nearly half of OB-GYNs saw those patients throughout their pregnancy (46.8%); more than half of Family Medicine providers had an ongoing (primary care and obstetric care) relationship with their pregnant patients (58.6%); whereas about one third of nurse-midwives provided routine pregnancy care only (37.5%), with the same number providing ongoing gynecologic care in addition to pregnancy care (36.5%).

### Telemedicine care

Telemedicine was currently practiced by a small percentage of providers (9.7%) and was reported as a logistically feasible future option for many more (44.2%), but not for the largest percentage of respondents (46.0%). Almost a quarter (24.9%) reported that telemedicine was an acceptable option, agreeing that the postpartum visit could be conducted as effectively by telemedicine as by an in-person visit. This belief did not significantly differ by specialty or years in practice. A chi-square test of independence was performed to examine the relation between region of provider and indication of support for telemedicine. The relation between these variables was significant (*X*^2^ (3, *N* = 224) = 11.32, *P* = .01), with greater provider-voiced support for telemedicine’s effectiveness in the West (42%) than in the South (28%), Northeast (21%), or Midwest (15%). For those disagreeing that telemedicine could be used to effectively conduct a postpartum visit, the primary barriers were inability to complete a pelvic exam (21.3%) or other physical exam, (e.g., vital signs) (35.7%). Many respondents noted that their position might change depending on the need for a physical exam; one offered, “I do pelvic exam on many of the patients, but telemedicine could work unless we identify a reason to come in for pelvic exam.” Those supportive of telemedicine as an effective alternative largely reported that telemedicine could capture the psychosocial evaluation and counseling elements of the postpartum checkup, although 14.5% of them still voiced reservations about maintaining the strength of the patient-provider relationship if appointments were conducted from a distance. An inter-rater reliability analysis found substantial agreement between the two raters after the first round of coding for this open-ended response (Kappa = 0.73, *P* < .001).

The mean appointment attendance rate was 75% for patients of providers who supported telemedicine approaches, and 81% for patients of providers who did not, (t(198) = 1.97, *P* = .05), illustrating that those with lower postpartum attendance rates, tend to report higher support for telemedicine. As one provider noted, “There is benefit to human touch and contact. There are also many nonverbal cues that could be missed when not viewing the total person. However, telemedicine beats no visit at all.”

## Discussion

Several guidelines for postpartum care exist [9–12], yet even for non-risky pregnancies and deliveries, providing comprehensive care during the limited time of a single postpartum visit is challenging. We sent a survey to 600 randomly-sampled U.S. postpartum care providers about their priorities for and the frequency with which they administer specific elements of postpartum care, as well as the feasibility and acceptability of telemedicine for postpartum care provision.

By contrasting self-reported priorities for care with the frequency by which providers report practicing that care, we can identify potential “tradeoffs” being made given competing demands on providers’ time. Identifying these tradeoffs allows us to think about how providers’ priorities and practices currently deviate from guidelines. We can see whether providers report not practicing care that is included in various postpartum care guidelines or whether they prioritize care that is currently absent from such guidelines. If providers are routinely administering care that they do not prioritize, such practices may just be part of their routine, even if they do not feel they are particularly important in terms of value. If providers prioritize elements of care that they do not routinely practice, these may represent elements of care that they find challenging to administer, elements they feel would best be performed by other care professionals, or elements they don’t perceive as normative – even if they think they should be. As the professional guidelines for postpartum care evolve, insights into competing demands in a single postpartum care visit can serve as a foundational step in understanding what elements of care under existing guidelines may require further examination or clarification, as well as which elements of care, not currently detailed in existing guidelines, warrant future, evidence-based inclusion.

Specifically, those elements of care at the extremes of prioritization and practice are worth examining further in order to inform recommendations about how to better prioritize and structure care under a call for an ongoing postpartum care process or what could, if anything, be removed from standard care. For example, the majority of providers reported that they routinely perform a pelvic exam at 6-weeks (as dictated by some guidelines), but actually placed considerably lower priority on that exam than other areas of care. This result suggests that the evidence-base of a routine pelvic exam may need to be re-examined as the guidelines for postpartum care evolve. A similar pattern was observed for counseling about resuming sexual activity. Time spent on such counseling may be at the request of the patient, but viewed as low value care on the part of the provider. There may be more efficient ways to communicate with patients about resumption of sexual activities, either through other clinical staff or through educational materials. Some patients may feel that a pelvic exam is necessary to “sign-off” on the resumption of sexual activities. If so, changing the norm for the pelvic exam may subsequently change expectations about the need for sexual activity counseling. In support of the supposition that providers priorities are concordant with clinical value, it is notable that the elements of care that were consistently highly practiced and prioritized across postpartum care providers are those with the strongest evidence-base for value, e.g. depression screening and addressing birth-related complications.

Intimate partner violence screening, which was highly prioritized by all specialties, was consistently underperformed compared to other highly prioritized clinical care (e.g., assessing C-section incision healing). The same was true to a lesser-degree for opioid and other substance use screening. Such ‘tradeoffs’ in care under time constraints may be the result of personal comfort level or experience, heuristics for prioritizing clinically pressing care that has a more immediate solution, or perceived professional norms. Family Medicine providers, for example, reported asking about substance use more frequently than other specialties, possibly because they are more likely to have long-term care relationships with their patients and feel more comfortable asking about socially stigmatized issues or have greater familiarity with their patient’s contextual circumstances. In transitioning to postpartum care as an ongoing process, providers and healthcare systems may wish to implement multiple touch points in such a way that clinically pressing care is addressed immediately and in-person, but other prioritized care, such as intimate partner violence or substance use screening, has a dedicated separate process in place to ensure that it doesn’t fall through the cracks. Professional organizations and healthcare systems may even wish to establish explicit norms about which providers conduct what type of care and at what timepoint to ensure comprehensive postpartum care is received.

The fact that our open-ended questions identified an additional category of priority care provision (transitioning to parenthood) that was not covered by existing guidelines, further highlights the constraints of providing all desirable elements of care during a single appointment. Moreover, many providers reported that appreciably fewer of their patients attended postpartum-care than scheduled it, compounding the issue of limited time available for postpartum care. Transportation assistance and in-home visits have been traditionally used to overcome access barriers [14–17], yet can be expensive to scale. Telemedicine has been shown to improve quality of care and access to health services [18, 19], particularly for providing postpartum psychosocial care, and was supported as a replacement for postpartum care by almost a quarter of respondents.

There were caveats to telemedicine support, however. Providers suggested that while most elements of a physical exam (e.g., pelvic exam or some contraceptive method provision) require in-person care, many of the highly prioritized psychosocial assessments could be performed virtually, either through telephone or videoconferencing. Some providers voiced concern that telemedicine could negatively impact a patient’s comfort with disclosure or the provider’s ability to detect subtle signs of psychosocial risk factors, such as depression. There is evidence that telemedicine-based interventions are feasible for identifying and treating mental health issues [20–22]. However, the evidence is less clear on the cost-effectiveness of telemedicine, both for mental and physical health monitoring and intervention [19, 23–25]. Given the time and effort required to coordinate video conferencing between patient and provider (as opposed to telephone contact), further investigation is warranted into both cost effectiveness and the relative rates of disclosure with various configurations of telemedicine. Other forms of distance care, e.g. evidence-based mobile health apps or other remote monitoring [26, 27], may also offer a more accessible way to delivery ongoing postpartum care, particularly for elements of care that do not require facetime with a provider, e.g. education about resuming sexual activities or diet and weight trajectory. These forms of care delivery may also have more appeal for those patients whom providers suspect are not attending in-person care because they “feel fine” or do not view the in-person visit as particularly important.

It is notable that in our open-ended questions, no respondents reported on the role of financial reimbursements in their priorities or decision making. This could be the result of how our questions were structured (focusing primarily on appropriate clinical care provision, as opposed to structural constraints or influences on care). However, any U.S. postpartum policy change, including implementation of distance care, would need to consider the different reimbursement systems that providers are operating under, e.g. bundled payments for maternity care.

A last but vital consideration for distance care is existing health inequities. Women with public insurance and African-American women are at considerably higher risk of severe postpartum maternal morbidity and mortality. Results showing that postpartum visit attendance is less common among women with public insurance were replicated here in providers’ self-reports. Currently, 94% of reproductively-aged women own a smartphone across sociodemographic groups [28] and early research suggests that African-American women may actually be more reliant on digital sources for accessing health information [29]. As such, evidence-based mobile apps, with provider-oversight, may be the most promising alternative for engaging and monitoring postpartum risk among a diverse population of peripartum women and could even help bypass structural racism or care access inequities if they are implemented thoughtfully.

### Limitations

Although respondents were randomly drawn from national lists, our sample size was small and tended to reflect providers with greater clinical experience (the mean years of practice was 25.0 ± 11.8). As such, the generalizability of our findings is limited. While the consistency in our sample of both priorities and practice across specialties could reflect a consensus among practitioners, it is also possible that our sample is biased towards those in their respective specialties who have strong opinions regarding postpartum care practices; therefore, caution should be exercised in considering any differences by specialty. Moreover, our response rate was higher from nurse-midwives that from other specialties, even though nurse-midwives do not provide the majority of postpartum care in the US. We suspect that this overrepresentation was due, in part, to differences in accuracy and completeness of the professional membership lists from which our survey recipients were selected.

It is also possible that social desirability may have played a role in survey responses, resulting in misidentified discrepancies. While the anonymity of the survey return process was designed to minimize this risk, it is possible that respondents overreported the frequency with which they performed certain care tasks, particularly those that they reported as important. Alternately, they may have reported that tasks were more important than they actually felt was true. If the former were the case, then our analysis would be conservative with respect to the amount of “inefficiencies.” If the latter were true, we would expect to see ceiling effects (elements being rated as “very important” across the board), which was not the case in our data. From the detail provided in our open-ended responses, we assume that considerable time and thought went into the majority of responses we received.

Lastly, we were limited by asking about general postpartum care priorities and practice. We did not ask about familiarity with various guidelines or the application of those guidelines under specific clinical circumstances. This was partly motivated by the fact that guidelines are directed to general care and partly to minimize survey burden. However, in practice, any element of postpartum care may be given more or less priority or selectively performed by providers depending on the specific patient. Moreover, the value of any given element of care may depend on the patient. While we did not ask about providers’ perceptions of patients’ priorities, it is possible that patient expectations may, in part, drive the care they receive at their postpartum visit. For example, the priority assigned to education about resuming sexual activity by our respondents was fairly low, on average, yet a provider may place greater importance on offering this service to a specific patient if it will notably alleviate that patient’s anxiety. More generally, providers may routinely opt to perform care that may not be considered clinically necessary, such as the routine pelvic exam, if it will offer a patient reassurance about their healing process. The less prioritized pelvic exam may also be appropriately performed for a given patient if, for example, a provider is seeing a patient with a postplacental IUD. This granular level of patient-specific assessment is part of the art of medicine, which cannot be fully dictated by guidelines. The results of this survey offer foundational research to inform thinking about guideline clarity on the part of professional organizations and structuring care to better adhere to guidelines, when appropriate, for healthcare systems. They also offer food for thought for providers who may wish to question elements of care that are unconsciously ingrained in their routine; proactively deciding to trade some off for care they may prioritize but not currently practice.

## Conclusion

With new ACOG recommendations, health care providers must determine how to prioritize care delivery and engage patients in postpartum care attendance. Such guidelines can be helpful if they are clear and unanimous, but risk causing confusion if they are not. Results from this survey suggest that providers largely provide the care that they prioritize most. However, given that the reported mean time spent with a patient in a postpartum care visit is less than 25 min, time constraints may be a considerable barrier to providing all the care that they prioritize, presenting competing demands in their care provision. The results of this survey demonstrate the tension that the current structure of a single postpartum care visit creates for time-constrained providers, during which they cannot always provide comprehensive care. Providers’ priorities and practices also offer a starting point for gathering data on the perception of value of elements of care that are not currently included in various guidelines, although a holistic approach would necessarily include the needs and priorities of the patients themselves.

As postpartum care becomes conceptualized as an epoch rather than an episode, guidelines from professional organizations that offer a clear ranking, distribution, and even recommended delivery mode of postpartum care responsibilities could minimize competing demands in providing critical postpartum care services. Our findings suggest that professional organizations should consider the practices and priorities of their constituency as they continue to develop guidelines to address postpartum needs. We also call on providers (and their respective healthcare systems) to reconsider practices that may be ingrained behavior, but not reflective of their priorities, designing new workflow for those elements of care that may be practiced less often than is desired.

## Supplementary information

**Additional file 1.**

## Data Availability

The survey questions are available in Supplemental materials (Additional File S1) and the dataset created during the current study is available from the corresponding author on reasonable request.

## Abbreviations

AAFP: The American Academy of Family Physicians
ACOG: The American College of Obstetricians and Gynecologists
ACNM: The American College of Nurse-Midwives
AMA: The American Medical Association
OB-GYN: Obstetrician-gynecologist
