# Supplementary material for: Competing demands in postpartum care: a national survey of U.S. providers’ priorities and practice
Source: BMC Health Serv Res. 2020 Apr 6;20:284. doi: 10.1186/s12913-020-05144-2 (PMC7137294; doi:10.1186/s12913-020-05144-2)
Supplement: Supplementary file 1 — Additional file 1. [file 12913_2020_5144_MOESM1_ESM.pdf]

HEALTHCARE PROVIDER OPINIONS ON POSTPARTUM CARE IN THE U.S.

These questions are the only open-ended questions. The rest of the survey is multiple choice. If you would like to take this survey online, you may scan the QR code above or use the link provided on the cover page.

1 **What are the main reasons a patient *should* attend a 6-week postpartum visit** with their OB/GYN, midwife or other pregnancy care provider?

Please provide as much detail as possible in whatever format you prefer.

2 **What are the main reasons a patient *does not* attend a 6-week postpartum visit** with their OB/GYN, midwife or other pregnancy care provider?

Please identify any factors that could play a role (e.g. demographic, geographical barriers, etc.).

3 **What are some solutions for overcoming barriers to attending postpartum care?**

Please provide as much detail as possible in whatever format you prefer.

4 **How *important* is it to address each of the following** at an in-office 6-week postpartum visit?

	Not at all	Slightly	Moderately	Very	Extremely
Vaginal birth complications	☐	☐	☐	☐	☐
C-section complications	☐	☐	☐	☐	☐
Pelvic exam	☐	☐	☐	☐	☐
Pregnancy onset complications (e.g. hypertension)	☐	☐	☐	☐	☐
Non-pregnancy related chronic conditions (e.g. cardiovascular disease)	☐	☐	☐	☐	☐
Postpartum depression and other mental health	☐	☐	☐	☐	☐
Intimate partner violence and other safety issues	☐	☐	☐	☐	☐
Breastfeeding and other infant feeding issues	☐	☐	☐	☐	☐
Resuming sexual activities	☐	☐	☐	☐	☐
Contraceptive counseling and family planning	☐	☐	☐	☐	☐
Contraceptive provision	☐	☐	☐	☐	☐
Safe sleep for baby	☐	☐	☐	☐	☐
Healthy sleep for mom	☐	☐	☐	☐	☐
Weight trajectory and diet information	☐	☐	☐	☐	☐
Smoking	☐	☐	☐	☐	☐
Opioid and other substance abuse	☐	☐	☐	☐	☐
Transitioning to a primary care provider	☐	☐	☐	☐	☐
Other (please specify): ____________________	☐	☐	☐	☐	☐

5	**How *often* do you address each of the following** at an in-office 6-week postpartum visit? If you do any of the following at a different visit, please mark "not this visit."

	Never	Rarely	Sometimes	Often	Always	Not this visit
Vaginal birth complications	☐	☐	☐	☐	☐	☐
C-section complications	☐	☐	☐	☐	☐	☐
Pelvic exam	☐	☐	☐	☐	☐	☐
Pregnancy onset complications (e.g. hypertension)	☐	☐	☐	☐	☐	☐
Non-pregnancy related chronic conditions (e.g. cardiovascular disease)	☐	☐	☐	☐	☐	☐
Postpartum depression and other mental health	☐	☐	☐	☐	☐	☐
Intimate partner violence and other safety issues	☐	☐	☐	☐	☐	☐
Breastfeeding and other infant feeding issues	☐	☐	☐	☐	☐	☐
Resuming sexual activities	☐	☐	☐	☐	☐	☐
Contraceptive counseling and family planning	☐	☐	☐	☐	☐	☐
Contraceptive provision	☐	☐	☐	☐	☐	☐
Safe sleep for baby	☐	☐	☐	☐	☐	☐
Healthy sleep for mom	☐	☐	☐	☐	☐	☐
Weight trajectory and diet information	☐	☐	☐	☐	☐	☐
Smoking	☐	☐	☐	☐	☐	☐
Opioid and other substance abuse	☐	☐	☐	☐	☐	☐
Transitioning to a primary care provider	☐	☐	☐	☐	☐	☐
Other (please specify): ______________________________	☐	☐	☐	☐	☐	☐

6a Is telemedicine (remote care) a feasible option for you or your patients now or in the near future?

- ☐ **Yes,** telemedicine is already an option at our practice
- ☐ **Yes,** telemedicine would be an option in the future
- ☐ **No,** telemedicine would not be a feasible option at our practice

6b Could the 6-week postpartum visit be assessed ***as effectively*** by telemedicine as by an in-person visit?

- ☐ Yes
- ☐ No

Please explain why or why not.

7 In your opinion, which of the following would provide the ***most effective*** postpartum care?

- ☐ A visit within 1-3 weeks postpartum
- ☐ The standard visit at 6 weeks postpartum
- ☐ A visit later than 6 weeks postpartum
- ☐ Postpartum visits *only* for those with specific medical concerns
- ☐ Other (please specify):

For the following question, please only consider your patient population.

8a Out of 100 patients at your practice, how many ***schedule*** a postpartum visit?

____ out of 100

8b Out of 100 patients that schedule a postpartum visit, how many ***attend*** their visit?

____ out of 100

Please briefly explain how you came up with those estimates.

9a On average, how frequently do you interact with your pregnant patients?

- ☐ I see my pregnant patients primarily for the birth
- ☐ I see my pregnant patients routinely throughout their pregnancy
- ☐ I have an ongoing primary (or obstetric) care relationship with my pregnant patients
- ☐ Other (please specify):

9b On average, how many minutes do you spend with a patient at their postpartum visit?

____ minutes with a patient

HEALTHCARE PROVIDER OPINIONS

These final pages are demographic questions about you and your practice. The rest of the survey should only take another few minutes.

10 What best describes your current position as a healthcare provider?

- ☐ Certified Midwife
- ☐ Certified Nurse-midwife
- ☐ Nurse Practitioner
- ☐ Practicing Physician.........Specialty (& sub-specialty, if applicable): ________
- ☐ Medical Resident............Specialty: ________ Year (e.g. 3rd) ____
- ☐ Medical Fellow................Specialty: ________ Year (e.g. 3rd) ____
- ☐ Other (please specify): ________

11 How many years have you been practicing as a healthcare provider? Include all training years.

________ years

12 What is the zip code of the practice where you see the majority of your patients?

____

13 Approximately what percentage of your patients have Medicaid?

________ %

14 In your practice, who primarily provides 6-week postpartum care?

- ☐ A Physician
- ☐ A Nurse-midwife
- ☐ A Nurse Practitioner
- ☐ A Physician's Assistant
- ☐ Other (please specify): ________

15 Do you personally treat women in the postpartum period?

☐ Yes ☐ No

16 What is your gender?

- [ ] Male
- [ ] Female
- [ ] Other (please specify): ______

17 What would you describe yourself as?

- [ ] Black/African-American
- [ ] Asian
- [ ] Hispanic/Latino(a)
- [ ] White/Caucasian
- [ ] Native American
- [ ] Other (please specify): ______

18 What is your highest level of educational attainment?

- [ ] Associate's degree
- [ ] Bachelor's degree
- [ ] Master's degree
- [ ] Medical degree (MD/DO)
- [ ] Doctorate degree (Psy.D., Ph.D.)
- [ ] Other (please specify): ______

Thank you for taking the time to complete this survey. Your responses are incredibly valuable to our work.

If you have any additional comments, please write them here. Feel free to attach more pages.

If you have any questions or concerns, you may contact our research team at tamark@pitt.edu or the University of Pittsburgh Human Research Protection Office at irb@pitt.edu.
